# The Potential Value of Near Patient Platelet Function Testing in PCI: Randomised Comparison of 600 mg versus 900 mg Clopidogrel Loading Doses

**DOI:** 10.1155/2010/908272

**Published:** 2009-10-20

**Authors:** Alex R. Hobson, Zeshan Qureshi, Phil Banks, Nicholas Curzen

**Affiliations:** ^1^Cardiac Intervention Unit, Royal Bournemouth Hospital, Castle Lane East, Bournemouth, BH7 7DW, UK; ^2^Wessex Cardiothoracic Unit, Southampton University Hospital, Tremona Road, SO16 6YD, UK; ^3^Southampton University Medical School, Southampton University Hospital, Tremona Road, Southampton, SO16 6YD, UK

## Abstract

Whilst poor response to clopidogrel is associated with adverse outcomes uncertainty exists as to how (a) response should be assessed and (b) poor responders managed. We utilised VerifyNow P2Y12 and short Thrombelastography (TEG) to assess 900 mg doses in (i) initial poor responders to 600 mg and (ii) in a randomised comparison with 600 mg. Blood was taken before and six hours post clopidogrel in (i) 30 volunteers receiving 600 mg (poor responders received 900 mg > two weeks later) and (ii) 60 patients randomized 1 : 1 to 600 mg or 900 mg doses. Poor response was defined as TEG %Clotting Inhibition (%CIn) or VerifyNow Platelet Response Unit (PRU) reduction <30%. (i) Poor responders to 600 mg had greater PRU reduction (45.0 versus 20.1%, *P* = 0.03) and greater %CIn (22.9 versus −15.1%, *P* = 0.01) after 900 mg but (ii) there were no significant differences between the patient groups. Near-patient assessment of response to clopidogrel is feasible and clinically useful. Whilst ineffective on a population basis 900 mg doses increase the effect of clopidogrel in initial poor responders.

## 1. Introduction

The clinical value of dual antiplatelet therapy with aspirin and clopidogrel to reduce platelet-mediated cardiovascular events is now well established in patients with acute coronary syndromes and those receiving intracoronary stents. In the field of PCI, in particular, a large and expanding body of evidence indicates that periprocedural complication rates can be reduced by loading doses of clopidogrel given at least 6 hours before planned stenting. The superiority of 600 mg loading doses of clopidogrel over 300 mg is now widely accepted [1–4]. There remain several areas of uncertainty in relation to optimal clopidogrel therapy in PCI patients, however. First, whether the risk of periprocedural and 30 day events could be further reduced by a 900 mg loading dose. The current data are discrepant in this regard [5–7]. Second, it is clear that there is heterogeneity in the response of individual patients to clopidogrel and that poor responders are susceptible to both early events [8–10] and later stent thrombosis [11–14]. Taking these factors into account, there is a logical case to be made that all patients being treated with clopidogrel should have their platelet function assessed to ensure a therapeutic response with the intention of reducing their risk. This concept is undermined by the limitations of current options for platelet function testing. Tests that are considered to be the “gold standard” for assessment of platelet function are generally laboratory based and analyse the platelet in isolation, rather that providing the clinician with an impression of the effect of clopidogrel on the patient's tendency to clot. The high proportion of patients labeled as “poor responders” by these assays illustrates their limited value in the clinical arena. By contrast, clinically relevant near patient tests are few and there is uncertainty about their reliability. As a result global testing of patient responses to clopidogrel still does not occur, despite the knowledge that if it did it has the potential to reduce the incidence of both periprocedural myocardial infarction and stent thrombosis. 

In this study we have the following aims:

1. to investigate the incidence of poor response to clopidogrel 600 mg in healthy volunteers and to assess whether the response of these individuals could be modified by 900 mg loading;
2. to compare platelet function in patients being considered for elective PCI and randomised to either a 600 mg or 900 mg loading dose of clopidogrel.

These experiments employed two near patient tests of platelet function: (a) VerifyNow and (b) a previously described and validated modification of Thrombelastograph PlateletMapping [15]. The latter, “short TEG” provides an assessment of platelet function in whole blood in 15 minutes.

The overall hypothesis for these experiments was that near patient assessment of platelet function in individual patients is feasible and provides clinically valuable information that could be used to tailor clopidogrel therapy where response was deemed to be inadequate.

## 2. Methods

### 2.1. Study Population

Approval was obtained from the Southampton and South West Hampshire Research Ethics committee B prior to commencing the study. All subjects provided written informed consent.

### 2.2. Group A

30 volunteers were recruited and received a 600 mg loading dose of clopidogrel. Poor responders to this initial loading dose received a 900 mg loading dose of clopidogrel at least two weeks later. Individuals were excluded if they had taken antiplatelet medication or nonsteroidal anti-inflammatory medication within 14 days or if they had a history of peptic ulceration, bronchial asthma, or bleeding.

### 2.3. Group B

60 patients receiving a loading dose of clopidogrel prior to either elective PCI or coronary angiography with a view to proceed were recruited. They were randomised 1 : 1 to 600 mg or 900 mg loading doses of clopidogrel. All patients were established on aspirin 75 mg maintenance therapy for >28 days. Exclusion criteria were use of antiplatelet or anticoagulant medication other than aspirin in the preceding 28 days, known intolerance to clopidogrel, planned use of glycoprotein IIb/IIIa inhibitor, recent bleeding, major haematological disturbance, and known malignancy.

### 2.4. Sample Analysis

Blood tests were taken for Thrombelastograph PlateletMapping (Haemoscope Corp, Ill, USA) (TEG) and VerifyNow P2Y12 assays (Accumetrics Inc., CA, USA) immediately before and six hours after drug administration in Group A and prior to and one, two, six and 20–24 hours after drug administration (before administration of maintenance doses of clopidogrel) in Group B. In all subjects venesection was performed from the antecubital fossa and the first 2 mL of blood discarded. Blood was then drawn into 2 mL 3.2% sodium citrate Vacutainers for VerifyNow analysis and a 6 mL Lithium Heparin Vacutainer for TEG analysis. Samples were analysed according to manufacturer's instructions. The percentage reduction in VerifyNow PRU from baseline was calculated at each timepoint, with reduction of <30% considered to represent poor response to clopidogrel. TEG analysis was performed using TEG PlateletMapping (Haemoscope, Ill, USA) which incorporates three channels: a “thrombin channel” with maximal platelet activation achieved through reversal of heparin with heparinase and maximal thrombin stimulation with kaolin activation; a “fibrin channel” representing platelet independent clot formation (generated without thrombin generation by the addition of reptilase and factor XIIIa to a heparinised sample) and channels with reptilase, factor XIIIa and ADP stimulation “the ADP channel.”

For TEG samples the percentage platelet inhibition (%PIn) and percentage clotting inhibition (%CIn) were calculated as previously described with %PIn and %CIn of <30% considered to represent poor response [15, 16]. 

Briefly, the %PIn was calculated from the maximum amplitude of the TEG traces by comparing the ADP channel with maximal platelet activation in the thrombin channel whilst subtracting the effect of the fibrin channel [16] and the %CIn was calculated from the area under curve of the TEG trace by comparing the response to ADP with the response to thrombin [14]. 

In Group B analysis of results was performed by an individual blinded to the dose administered. 

Some patients received unplanned use of glycoprotein IIb/IIIa inhibitors (all Abciximab) or Bivalirudin during PCI. In these cases blood tests were not performed at any time after administration of Abciximab or within six hours of cessation of Bivalirudin infusion.

### 2.5. Clinical Endpoints

For Group B rates of major and minor bleeding, periprocedural myocardial infarction, stent thrombosis, and death were compared between the two groups.

### 2.6. Statistical Analysis

For Group A previous data suggest that a group size of 30 is required to identify 10 poor responders [15, 17]. Ten poor responders would be required to detect a 10% difference in platelet function with the higher dose with 80% power using paired, two-tailed *t*-tests. For Group B the study has been powered to detect a 25% relative difference in platelet inhibition with 80% power. Significance between the groups was determined using two-tailed, two group *t*-tests with a *P* value of <.05 considered to represent significance. Data are presented as the mean ± 95% confidence interval of the mean. Fisher's exact tests were used to determine differences between numbers of responders.

## 3. Results

### 3.1. Group A

#### 3.1.1. Baseline Demographics

15 males and 15 females were recruited. Age 33 ± 4 years. Two volunteers were smokers. Two individuals were withdrawn prior to study completion as they developed a prespecified exclusion criteria. Data from 28 volunteers was therefore analysed.

#### 3.1.2. Antiplatelet Effect

**VerifyNow**

Baseline mean PRU was 264 ± 16 compared to 106 ± 28 six hours after a 600 mg loading dose of clopidogrel. Mean % reduction in PRU was 59.9 ± 10.1%.

**Figure 1:**
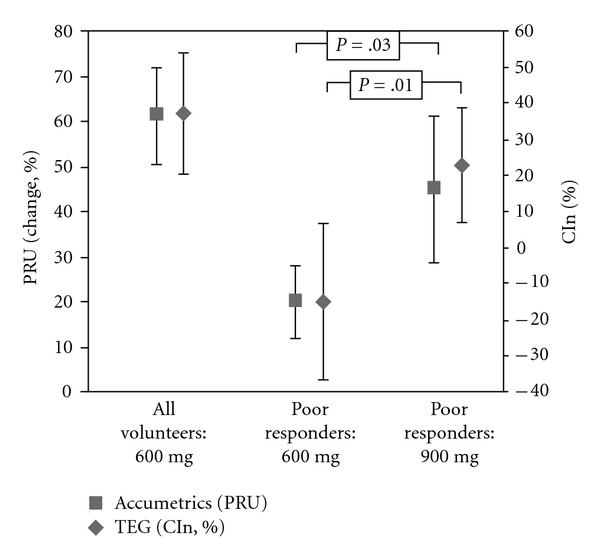
Change in PRU and %CIn 6 hours after clopidogrel therapy for (i) all volunteers taking 600 mg clopidogrel (ii) poor responders taking 600 mg clopidogrel (iii) poor responders taking 900 mg clopidogrel.

Five volunteers (17%) were classified as nonresponders by VerifyNow. Initial nonresponders had significantly greater reduction in PRU after a 900 mg loading dose of clopidogrel (45.0 versus 20.1%, *P* = .03). Four of the five (80%) nonresponders to a 600 mg dose were classified as responders after the 900 mg dose (Figure 1, Table 1).

**Thrombelastography**

Six hours after the initial 600 mg loading dose of clopidogrel %PIn was 48.1 ± 11.6, %CIn was 37.7 ± 15.9%. Nine volunteers (31%) were classified as nonresponders by both %PIn and %CIn. In nonresponders to the 600 mg dose %PIn (32.0 versus 9.9%, *P* = .01) and %CIn (22.9 versus −15.1%, *P* = .01) were significantly greater after 900 mg of clopidogrel. Of the nine nonresponders to 600 mg clopidogrel four (44%) were classified as responders after a 900 mg loading dose by both %PIn and %CIn (Figure 1, Table 1).

### 3.2. Group B

#### 3.2.1. Baseline Demographics

There were no significant differences between the 2 groups in terms of baseline demographics, haematological parameters or in procedure undertaken (Table 2). Four patients received Abciximab, one patient received Bivalirudin and 18 patients were discharged prior to the 20–24 hour timepoint. Data was therefore analysed from 87% of the planned datapoints (100% at baseline, one and two hours, 92% at the six hour timepoint and 70% at the 20–24 hour timepoint).

#### 3.2.2. Clinical Endpoints

There were no significant differences between the groups in terms of procedural complications. There were no major bleeds and no acute stent thromboses. There was one access site haematoma (in the 900 mg group). There was no difference in periprocedural myocardial infarction (17% in the 600 mg group and 20% in the 900 mg group).

#### 3.2.3. Antiplatelet Effect

**VerifyNow**

Whilst there was no difference in % reduction in PRU between the two groups at the one and two hour timepoints, there was a trend towards greater reduction at six hours (55.3 ± 9.1 versus 37.2 ± 15.8, *P* = .06) and significantly greater reduction by 24 hours (60.9 ± 9.1 versus 37.9 ± 17.0, *P* = .04) in the 900 mg group (Table 3).

There were significantly fewer poor responders in the 900 mg group at the six hour timepoint (Table 4).

**Thrombelastography**

Whilst the %PIn and %CIn were greater in the 900 mg group at all timepoints there were no significant differences between the two groups. There was a trend towards greater %PIn in the 900 mg group at six hours (45.9 ± 8.4 versus 33.2 ± 8.9, *P* = .06) and at 24 hours (37.8 ± 7.7 versus 24.7 ± 7.1, *P* = .06) (Table 3). There were no differences between the two groups in the number of poor responders at any timepoint (Table 4).

## 4. Discussion

There is increasing evidence that poor response to clopidogrel post PCI correlates with subsequent ischaemic events and even death [3, 4, 8–10, 18]. Furthermore, studies have specifically suggested that relative hyporesponsiveness to clopidogrel increases the risk of Stent thrombosis [11–14]. However, at present responses are not routinely monitored. This is largely due to (a) the expense and complexity of laboratory based assays of platelet function; (b) difficulties in determining the threshold for poor response with poor positive predictive values; (c) uncertainty in how to manage poor response. 

There is now evidence that repeated loading doses [19]; increased maintenance doses of clopidogrel [19, 20]; and changing to Ticlopidine [19] or Prasugrel [21] improve the observed antiplatelet response and can decrease the rate of poor responders to clopidogrel by 60 [20] to 78.9% [19] but as yet little direct evidence that these strategies improve clinical outcomes post PCI. However, recently Bonello et al., using a strategy of VASP guided clopidogrel loading prior to PCI with up to 3 further 600 mg clopidogrel loading doses administered to obtain adequate response did, in addition to improving response to clopidogrel, achieve significantly improved MACE rates at 30 days without increased bleeding [22].

However, 900 mg loading doses of clopidogrel could increase both the speed and extent of the antiplatelet effect and decrease the number of poor responders but previous studies have shown discrepant results. The ALBION study suggested an increased effect with 900 mg doses [5]. However ISAR-CHOICE found no differences in effect and no differences in levels of the active metabolite of clopidogrel suggesting that absorption or metabolism of clopidogrel may be a limiting factor [6]. Price et al. also showed no difference in the magnitude of response or the time to maximal response with VerifyNow P2Y12 assays [7].

We have compared the antiplatelet effects of 600 mg and 900 mg loading doses of clopidogrel using VerifyNow and TEG, two rapid reliable tests of response to clopidogrel and platelet reactivity whilst on clopidogrel that are suitable for near patient clinical use. Both tests have been shown to correlate with optical aggregation, the current “gold standard” for measuring platelet activity [16, 23, 24]. In addition we have shown that both assays identify poor response to clopidogrel in patients with previous stent thrombosis [14]. Using these techniques we have demonstrated that near patient testing is feasible and provides individual patient data on response to clopidogrel in a time dependent manner. Further, these tests can both detect poor responders and also reassess responses after increased dose to determine if the response has become adequate. These data also demonstrate that whilst 900 mg clopidogrel loading doses do not significantly enhance platelet inhibition compared to 600 mg doses in the general patient population, selective and individualised dose adjustment in poor responders improves the level of response and reduces the number of poor responders. 

This study has some limitations. We did not correlate the results of our two near patient assays with laboratory based assays. In emergencies such as primary angioplasty this strategy would also be inappropriate and the role of “top up” doses in this context should be explored. In addition, this study was not designed to investigate long term clinical outcomes.

If these techniques and this approach of individualized therapy is to be incorporated into routine clinical practice large clinical outcome trials are obviously required. We do believe that in order to maximize the chances of these techniques becoming widespread these trials should be performed using rapid, simple near patient assays.

## 5. Conclusions

We have shown that near patient assessment of response to antiplatelet therapy with both VerifyNow and short TEG is feasible and could be used to tailor clopidogrel therapy where responses are deemed to be inadequate. Whilst there remain contentious issues, particularly in determining the threshold for “poor response” we believe that there is a clear role for universal near patient assessment of response to antiplatelet therapy and that soon it may become unethical to manage high risk individuals without it.

## Figures and Tables

**Table 1:** The %PIn, %CIn, and % Change in PRU of (a) all healthy volunteers 6 hours post 600 mg clopidogrel, (b) poor responders to 600 mg: 6 hours post 600 mg clopidogrel, (c) poor responders to 600 mg: 6 hours post 900 mg clopidogrel. Data presented as mean ± 95% confidence interval of the mean (numbers analysed).

	All Volunteers	Initial Poor Responders (to 600 mg)		
		Response to 600 mg	Response to 900 mg	*P*
%PIn	48.1 ± 11.6 (28)	9.9 ± 9 (9)	32.2 ± 16.3 (9)	.01
%CIn	37.7 ± 15.9 (28)	−15.1 ± 21.6 (9)	22.9 ± 15.9 (9)	.01
% Change in PRU	59.9 ± 10.1 (28)	20.1 ± 8.2 (5)	45.0 ± 16.3 (5)	.03

**Table 2:** Baseline demographics and haematological parameters in PCI patients randomized to 600 mg and 900 mg clopidogrel loading doses.

	600 mg group	900 mg group	*P* value
Age	65 ± 3.8	65.2 ± 3.7	.92
% male	70%	63.30%	.78
Weight	81.8 ± 5.6	81.3 ± 5.5	.86
Aspirin dose	80.4 ± 11.3	81.3 ± 11.4	.88
PCI performed	16 (53%)	19 (63%)	1.00
No sig. coronary disease	4 (13%)	1 (3%)	.35
Smokers	6 (20%)	1 (3%)	.10
Diabetics	6 (20%)	2 (7%)	.25
Haematoma	0	1 (3%)	1.00
Enzyme rise	5 (17%)	6 (20%)	.72
Hb	140 ± 5.7	139 ± 5.7	.86
Plt count	235 ± 23.7	240 ± 23.7	.68
Haematocrit	0.41 ± 0.01	0.41 ± 0.01	.84
e GFR	70.4 ± 5.4	70 ± 5.3	.88
INR	0.99 ± 0.0	0.99 ± 0.0	.94

**Table 3:** Response to clopidogrel in PCI patients randomised to 600 mg and 900 mg loading doses. (PRU: reduction compared to baseline; %PIn: Percentage platelet inhibition due to clopidogrel; %CIn: Percentage clotting inhibition due to clopidogrel.)

	600 mg group	900 mg group	*P* value
PRU			

1 hour	10.4 ± 10.3	22.3 ± 9.1	.09
2 hour	35.9 ± 13.3	44.5 ± 9.5	.31
6 hour	37.2 ± 15.8	55.3 ± 9.1	.06
24 hour	37.9 ± 17.0	60.9 ± 9.1	.04

%PIn			

1 hour	13.0 ± 7.2	22.0 ± 7.6	.10
2 hours	26.5 ± 9.5	33.2 ± 8.5	.31
6 hours	33.2 ± 8.9	45.9 ± 8.4	.06
24 hours	24.7 ± 7.1	37.8 ± 7.7	.06

%CIn			

1 hour	4.0 ± 11.2	4.4 ± 12.4	.95
2 hours	19.8 ± 11.7	23.3 ± 12.1	.68
6 hours	25.8 ± 8.6	35.7 ± 8.7	.13
24 hours	18.1 ± 11.5	31.2 ± 10.2	.19

**Table 4:** The percentage of “poor responders” to clopidogrel in PCI patients randomized to 600 mg and 900 mg clopidogrel loading doses. Poor response is defined as: PRU: <30% reduction in Accumetrics PRU from baseline; %PIn: short TEG %PIn <30%; %CIn: short TEG %CIn <30%.

	600 mg group	900 mg group	*P* value
PRU			

1 hour	77 (23/30)	67 (20/30)	.57
2 hour	40 (12/30)	27 (8/30)	.29
6 hour	41 (11/27)	12 (3/26)	.02
24 hour	41 (7/17)	15 (3/20)	.07

%PIn			

1 hour	70 (21/30)	77 (23/30)	.77
2 hours	60 (18/30)	43 (13/30)	.44
6 hours	43 (12/28)	30 (10/28)	.40
24 hours	61 (11/18)	37 (7/19)	.51

%CIn			

1 hour	77 (23/30)	80 (24/30)	.77
2 hours	43 (13/30)	53 (16/30)	.45
6 hours	36 (10/28)	33 (9/27)	1.00
24 hours	37 (7/19)	55 (11/20)	.34
